# Supplementary material for: Aphid Resistance in Pisum Affects the Feeding Behavior of Pea-Adapted and Non-Pea-Adapted Biotypes of Acyrthosiphon pisum Differently
Source: Insects. 2022 Mar 8;13(3):268. doi: 10.3390/insects13030268 (PMC8955652; doi:10.3390/insects13030268)
Supplement: Supplementary file 1 [file insects-13-00268-s001.zip › insects-1600372-supplementary.pdf]

Table S1 Pisum genotypes used in this study

Genotype	Species	AMS Code	Origin	Variety type	ArPo28*	LSR1**
WT304	*Pisum fulvum*	P_AMS_DCG0570	ISRAEL		41.1	77.68
AeD99OSW- 49-5-7	*Pisum sativum*	P_AMS_DCG0661	FRANCE	Proteaginous	84.5	8.81
NEVE	*Pisum sativum*	P_AMS_VKL0164	FRANCE	Proteaginous	91.2	7.02
CAMEOR	*Pisum sativum*	P_AMS_DCG0251	FRANCE	Garden	104.8	24.83
CE101 = FP	*Pisum sativum*	P_AMS_RCG0228	FRANCE	Proteaginous	163.6	181.55
CHEROKEE	*Pisum sativum*	P_AMS_DCG0472	FRANCE	Proteaginous	191.2	287.52

*ArPo28 aphid count. Three first instar (L1) larvae (generation 1, G1) produced on *V. faba* were installed on each Pisum genotype. Ten days after the G1 installation, three L1 larvae (G2) were installed on a new *Pisum* plant of the same genotype. Then, 18 days after the installation of G2 aphids, all their offspring (G3) were collected and counted [13].
**LSR1 aphid count. Ten mixed-aged aphids were installed on each *Pisum* plant and all aphids (G1 adults and G2 offspring) were collected and counted three weeks later [13].
